# Long-term dietary (n-3) polyunsaturated fatty acids show benefits to the lungs of Cftr F508del mice

**DOI:** 10.1371/journal.pone.0197808

**Published:** 2018-06-01

**Authors:** Céline Portal, Valérie Gouyer, Renaud Léonard, Marie-Odile Husson, Frédéric Gottrand, Jean-Luc Desseyn

**Affiliations:** 1 Inserm, Université de Lille, CHU Lille, LIRIC – UMR 995, Lille, France; 2 CNRS, Université de Lille, UGSF – UMR 8576, Villeneuve d’Ascq, France; Public Library of Science, UNITED KINGDOM

## Abstract

**Introduction:**

The pro-inflammatory status of cystic fibrosis (CF) patients promotes pulmonary colonization with opportunist and pathogenic bacteria, which is favored by a sticky mucus. Oral supplementation with (n-3) long chain polyunsaturated fatty acids (LC-PUFA) has shown anti-inflammatory effects. The aim of this study was to demonstrate the positive effects of a long-term diet enriched in (n-3) LC-PUFA on the lungs of Cftr F508del mice.

**Materials and methods:**

Breeding CftrΔF508del/+ mice received a control diet or a diet enriched in (n-3) LC-PUFA for 5 weeks before mating, gestation and lactation. After weaning, the offspring were given the same diet as their mother until post-natal day 60. The effects of (n-3) LC-PUFA supplementation on the lungs were evaluated in homozygous Cftr F508del mice and their wild-type littermates after acute lung inflammation induced by *Pseudomonas aeruginosa* lipopolysaccharide (LPS) inhalation.

**Results:**

(n-3) LC-PUFA enrichment of mothers contributes to enrichment of mammary milk and cell membrane of suckling pups. Cftr F508del mice exhibited growth retardation and lung damage with collapsed alveoli, hyperplasia of bronchial epithelial cells and inflammatory cell infiltration. The (n-3) LC-PUFA diet corrected the growth delay of Cftr F508del mice and decreased hyperplasia of bronchial epithelial cells. Besides decreasing metaplasia of Club cells after LPS inhalation, (n-3) LC-PUFA modulated lung inflammation and restricted lung damage.

**Conclusion:**

Long-term (n-3) LC-PUFA supplementation shows moderate benefits to the lungs of Cftr F508del mice.

## Introduction

Cystic fibrosis (CF) is a fatal hereditary disease resulting from mutation in the CF transmembrane conductance regulator (*CFTR*) gene. The disease is most common in Caucasian populations (1 in 2500 live births). Amongst the plethora of mutations reported in the gene, deletion of the phenylalanine residue in amino acid position 508 is the most common (>65%). Dysfunction of the CFTR protein results in accumulation of viscous mucus in the lungs, intestine and pancreas. Bacterial infection of the airways is the main hallmark of the disease. CF patients cannot clear opportunistic pathogens such as *Pseudomonas aeruginosa*, which is responsible for progressive loss of lung function and remains the leading cause of morbidity and mortality in CF patients. The link between the CFTR defect and increased susceptibility to *P*. *aeruginosa* infection may be explained by the overly viscous mucus in the airways reducing bacterial clearance and immune cell function fighting the bacteria and excessive inflammation which starts early during infancy [1,2] and even during fetal life [3]. Essential fatty acids deficiency was reported in CF and seems due to defects in fatty acid metabolism [4,5] and fat malabsorption which is a key feature in more than 80% of CF patients [6], especially concerning essential omega-3 (n-3) long-chain polyunsaturated fatty acids (LC-PUFA) such as eicosapentaenoic acid (EPA) and docosahexaenoic acid (DHA) [7]. This results in an imbalance between (n-3) LC-PUFA and (n-6) LC-PUFA in cell membranes consistent with overall suppression of the anti-inflammatory pathway mediated by (n-3) LC-PUFA metabolites and exacerbation of the inflammatory pathway mediated by metabolites of arachidonic acid (AA), which is the most common (n-6) LC-PUFA [8]. Clinical trials have attempted to demonstrate that dietary supplementation with (n-3) LC-PUFA may improve patient outcomes. The most recent review concluded that (n-3) LC-PUFA supplements may provide some benefits for CF patients with relatively few adverse effects, but the evidence is currently insufficient to draw firm conclusions or recommend the routine use of these supplements [9].

Several mouse models with a mutated *Cftr* gene have been created and characterized. The occurrence of LC-PUFA deficiency in CF mice, including the Cftr F508del genetic model, is not consistent in the literature, possibly due to the genetic background of the mice, the *Cftr* mutation and age [10]. In Cftr^–/–^ mice, we have previously reported that supplementation for 5 weeks with a diet enriched in DHA and EPA corrected the LC-PUFA imbalance and improved the resistance of mice to experimental *P*. *aeruginosa* infection [11,12]. Less is known in Cftr F508del mice but the DHA/AA ratio was reported to increase in tissues when adult mice received a low dose of DHA for 6 weeks [10]. In this study we aimed to better characterize the lungs of Cftr F508del mice and to try to show the beneficial effects of a long-term diet enriched in EPA+DHA on lung damage after an acute lung inflammatory challenge.

## Materials and methods

### Mice and diets

Male and female Cftr^tm1EUR^ mice (Cftr F508del [13]) were provided by the French Center of Transgenesis, Archiving and Animal Models in Orléans (CDTA, Orléans, France) are named thereafter CftrΔF508. Heterozygous mice for the CftrΔF508 mutation (FVB genetic background) were housed in our specific pathogen-free animal facility. Food and drinking water were provided *ad libitum*. To prevent intestinal occlusion in homozygous mice, all mice received polyethylene glycol 4000 (44.4 g/L) in the drinking water. Heterozygous male and female CftrΔF508 mice were fed with a control diet (AING-93G) or an isocaloric and isolipidic diet enriched in (n-3) LC-PUFA [12,14] for 5 weeks before mating. Female heterozygous CftrΔF508 mice were given the same diet during gestation and lactation. After weaning, the offspring were given the same diet as their mother until post-natal (PN) day 60. At PN60, male and female homozygous CftrΔF508 offspring and their wild-type (WT) littermates were studied. All procedures were in accordance with the French Guide for the Care and Use of Laboratory Animals and with the guidelines of the European Union. The protocol was approved by the Ethics Committee of Lille (CEEA 462012).

### Milk, lung and liver tissue composition

Lipids were extracted from the lungs and liver of adult mice at PN60, and from lungs, liver and stomach content of pups between PN1 and PN3. Briefly, samples were homogenized in methanol/chloroform (2/1, v/v) before centrifugation at 10000 x *g* for 2 min. The supernatant was then submitted to three steps of water/chloroform (1/1, v/v) extraction. The chloroform phase was dried under a nitrogen flow. Samples were then esterified to the more volatile methyl esters by the methanol·BF3 method at 45°C for 45 min and separated by capillary gas chromatography performed on a BPX70 SGE column (30 m in length, 0.25 mm internal diameter) using a temperature gradient starting at 68°C and reaching 70°C at 2 min, 190°C at 14 min, 198°C at 22 min, 218°C at 24 min and 225°C at 45 min. Identification and quantification of fatty acids was performed by injecting authentic standard solutions (Supelco-47085-U; Sigma Aldrich, France).

### Lipopolysaccharide (LPS)-induced lung inflammation

LPS-induced lung inflammation was performed at PN60 by intranasal inhalation of *P*. *aeruginosa* LPS. After ketamine (100 mg/kg)-xylazine (10 mg/kg) (2:1) anesthesia, 50 μL of *P*. *aeruginosa* LPS (1 mg/mL, Sigma, France) was placed on the edge of the nostril and was inhaled by the mouse. Control mice were given sterile phosphate-buffered saline (PBS) instead of LPS. Mice were killed 24 h later by intraperitoneal injection of 11 mg of sodium pentobarbital (Ceva, France).

### Tissue collection

After euthanasia, the lungs were isolated and rinsed in PBS. The lobes were separated according to their use: the left lobe was fixed by immersion in 4% paraformaldehyde (PFA) for 18 h and the right lobes were frozen in liquid nitrogen and kept at –80°C until use.

### Histologic score

The PFA-fixed lobe of the lung was dehydrated by successive baths in ethanol of increasing strength and toluene. Dehydrated tissues were embedded in paraffin. After dewaxing and rehydration of 5 μm sections, hematoxylin/eosin (HE) and periodic acid-Schiff (PAS) staining was performed to evaluate lung damage using a score adapted from Bilsborough *et al*. [15]. The seven criteria are presented in Table 1. The presence of mucus plugs was defined as the presence of at least one bronchus with mucus material filling at least the half of the lumen on one of the sections scored for the same lung. All criteria were scored in HE sections, excepted for mucus plugs which were evaluated on PAS sections. For each mouse, the histologic score was established blindly by two independent examiners on 3–14 sections representative of the whole lung. The total histologic score for each animal is the sum of the score for each criterion divided by the number of sections scored. For the presentation of the results, the scores for infiltration of inflammatory cells and specific perivascular and peribronchial infiltration of inflammatory cells were added and expressed as total inflammatory cells.

**Table 1:** Histological score of lung damage.

Points	0	1	2	3	4
% of lung injury	0–20%	20–40%	40–60%	60–80%	80–100%
% of collapsed or irregular alveoli	0–20%	20–40%	40–60%	60–80%	80–100%
Hyperplasia of bronchial epithelial cells	None	Mild	Severe	Severe with important thickening	–
Metaplasia of Club cells	None	Mild	Severe	–	–
Plugs of mucus	Absence	Presence	–	–	–
Infiltration of inflammatory cells	Absence	Presence	–	–	–

### Enzyme-linked immunosorbent assay (ELISA) of cytokines

For homogenate preparation, frozen lungs were homogenized with a disperser (Ultra-Turrax) in lysis buffer containing 0.1 M dithiothreitol, 0.01% Nonidet P-40 and protease inhibitor cocktail (Complete mini EDTA-free; Roche, Switzerland), and then centrifuged at 1000 x *g* for 10 min. Supernatants were aliquoted and kept at –80°C until use. A mouse CXCL1/KC DuoSet kit (R&D Systems, USA) was used to determine the keratinocyte chemoattractant (KC) concentration in lung homogenates, and interferon-gamma (IFNγ) and tumor necrosis factor alpha (TNF-α) concentrations were quantified with ELISA Ready-SET-Go kits (eBioscience, USA). Samples were assayed according to the kit instructions. Cytokine concentrations were compared with the quantity of total proteins in lungs determined with a bicinchoninic acid (BCA) protein assay kit (Pierce, USA) according to the manufacturers’ instructions.

### Quantitative real-time polymerase chain reaction (PCR)

Total RNA was isolated from frozen lung tissue using TriReagent as described previously [14]. Two micrograms of RNA were reverse transcribed to synthesize cDNA using 0.2 U of MMLV reverse transcriptase (Promega, USA) and random hexamers according to the manufacturer’s instructions. Amplifications using 18S rRNA as an internal control (TaqMan Ribosomal RNA Control Reagents; Applied Biosystems, USA) were performed as described previously [14]. The sequences of primers and probes used to analyze *PPARα* and *PPARγ* expression were selected using Primer3 Output program technology (MIT) and were as follows: for *PPARα* (126 bp length): forward primer 5'-CGTTTTCACAAGTGCCTGTCT-3', reverse primer 5'-CGAATCTTTCAGGTCGTGTTC-3' and probe 5'-GCAATTCGCTTTGGAAGAATGCCA3'; and for *PPARγα* (122 bp length): forward primer 5'-ACCCAATGGTTGCTGATTACA-3', reverse primer 5'-ATGAGGCCTGTTGTAGAGCTG-3' and probe 5'-TGAAGCTCCAAGAATACCAAAGTGCGA-3'. Amplifications were performed in triplicate with a 7500 Applied System (Applied Biosystems, USA). For each sample, the ratio of amplification was calculated as 2^-(Ct_mean_ target gene – Ct_mean_ 18S).

### Data and statistical analysis

The Wilcoxon-Mann-Whitney test was used to compare unpaired data. Pearson’s Chi-square test was used to analyze mucus plug frequency data. The inter-observer agreement for lung scoring was calculated using the *κ* values from the Cohen’s Kappa test. All data were analyzed using StatXact 6.0 (Cytel Studio, Cambridge, MA) for exact nonparametric inference. A p-value ≤ 0.05 was considered statistically significant.

## Results

### Early incorporation of (n-3) LC-PUFA in tissues

**Fig 1:**
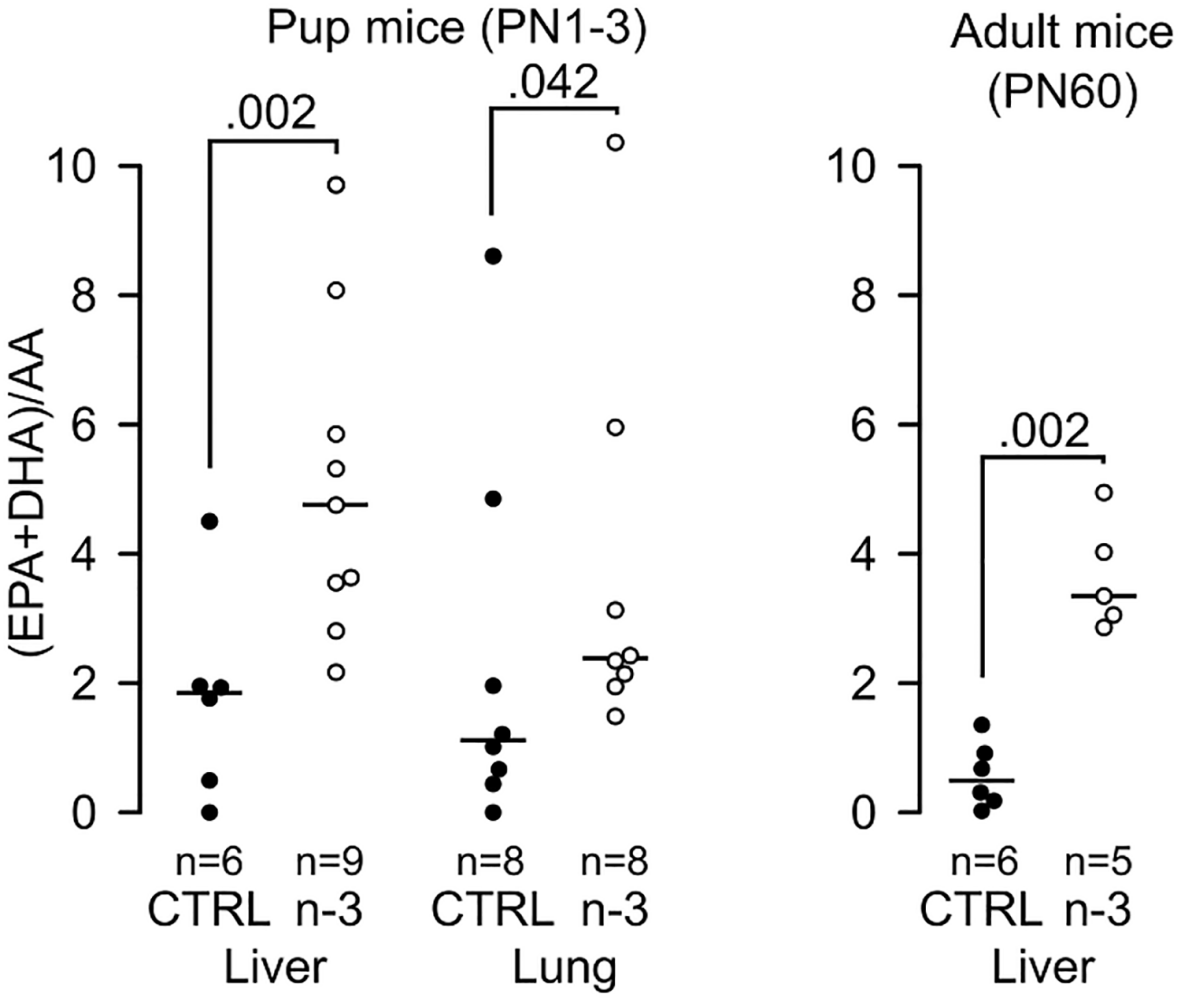
Early (n-3) LC-PUFA consumption improves fatty acid incorporation in tissues of CftrΔF508 mice. (n-3) LC-PUFA incorporation was quantified in pup liver and lungs at post-natal (PN) day 1–3 and in adult liver on PN60 (5–9 mice/group). CTRL: control diet group; n-3: (n-3) LC-PUFA diet group; WT: wild-type mice; ΔF508: CftrΔF508 mice.

(n-3) LC-PUFA incorporation was assayed in the milk (from the stomach), lungs and liver from CftrΔF508 pups (between PN1 and PN3) and in the lungs and liver from CftrΔF508 adult mice (PN60). The ratio of (DHA+EPA)/AA was 5.8-fold higher in milk from suckling pups’ stomachs and 6.1-fold higher in adult lungs compared to mice fed the control diet. The (n-3) LC-PUFA content of the studied tissues of pups (liver and lungs) and adult mice (liver) reflected the diet (Fig 1). This shows that (n-3) LC-PUFA enrichment of pregnant mice contributes to enrichment of the cell membrane with (n-3) LC-PUFA in suckling pups.

### CftrΔF508 mice exhibited a growth delay which was corrected by (n-3) LC-PUFA in males

**Fig 2:**
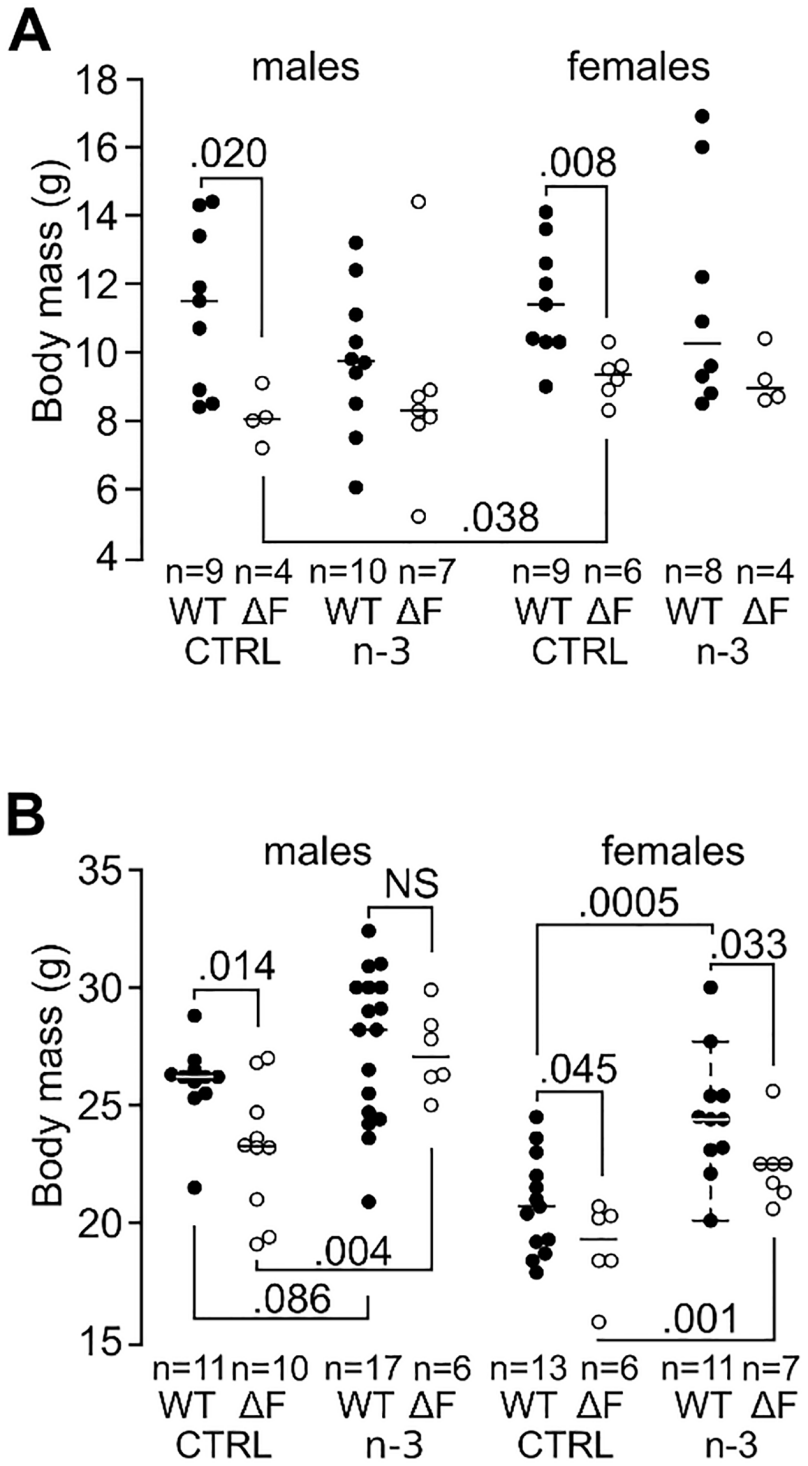
Early (n-3) LC-PUFA consumption corrects growth retardation in male CftrΔF508 mice. Body mass of CftrΔF508 (ΔF) and brother-sister wild-type (WT) mice at weaning **(A)** and adulthood **(B)** (4–10 pup mice and 6–17 adult mice/group). NS: not significant; CTRL: control diet group; n-3: (n-3) LC-PUFA diet group; WT: wild-type mice; ΔF508: CftrΔF508 mice.

At PN21, male and female CftrΔF508 mice fed control diet showed growth retardation compared to WT mice (–29%, *P* = 0.020 and –19%, *P* = 0.008, respectively, Fig 2A) and WT mice grew less under the (n-3) LC-PUFA diet with a similar body mass for the two genotypes (Fig 2A). At adulthood, growth retardation persisted in CftrΔF508 mice fed the control diet compared to WT mice albeit much lower than at PN21 (–11%, *P* = 0.014 and –10%, *P* = 0.045 for males and females, respectively, Fig 2B). The (n-3) LC-PUFA diet was associated with an increased body mass in CftrΔF508 mice (+16.3%, *P* = 0.004 and +16.6%, *P* = 0.001 for males and females, respectively), which became similar to WT mice in males (*P* = NS) while females remained lighter (*P* = 0.033). Together, these data show that (n-3) LC-PUFA significantly reduces growth retardation in CftrΔF508 mice.

### Lungs of CftrΔF508 mice are more susceptible to LPS challenge and (n-3) LC-PUFA diet shows slight improvement after LPS challenge

**Fig 3:**
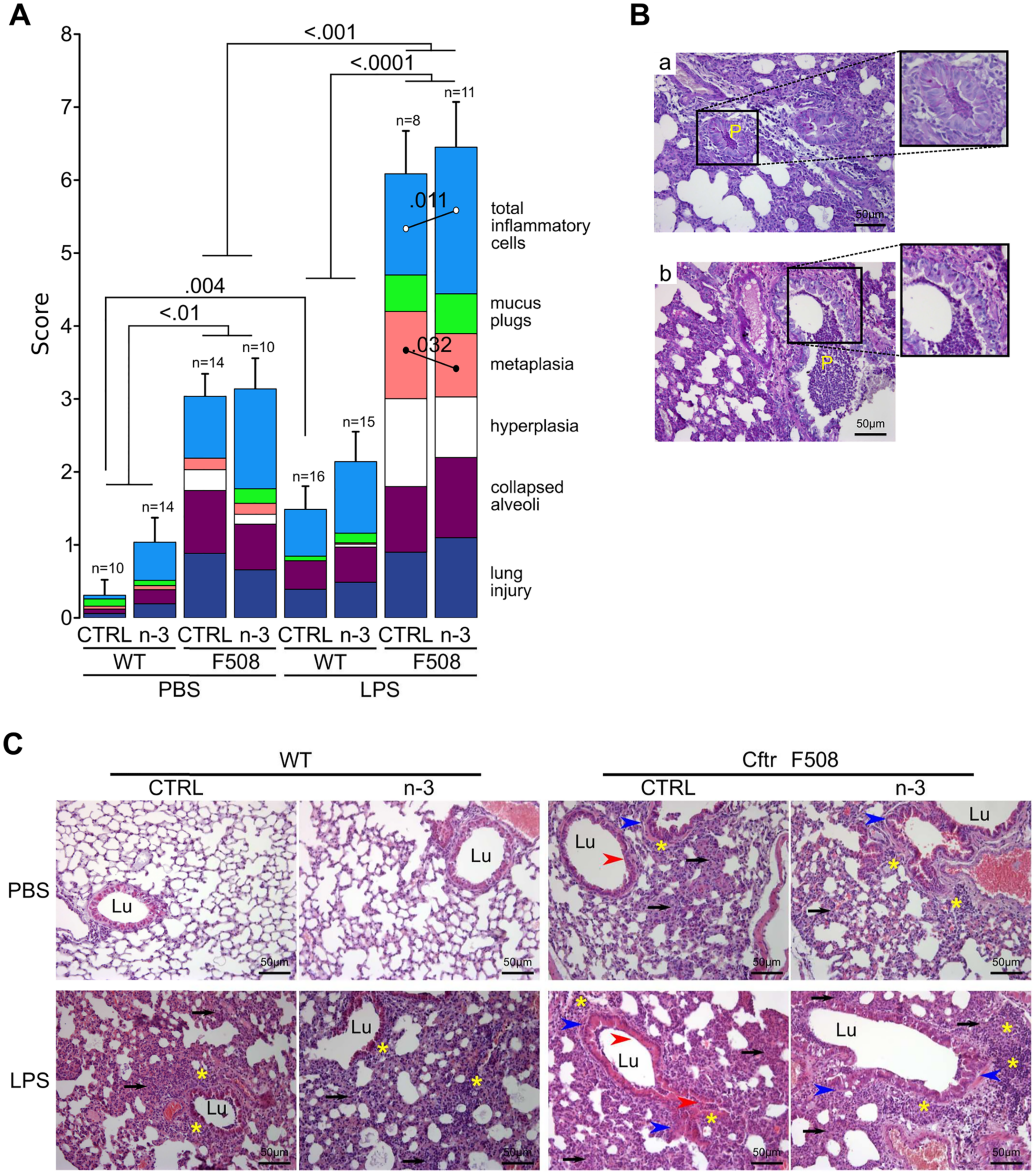
Histologic score for lung damage. **(A)** Total histologic score is the sum of the score for each criterion (lung injury areas, collapsed alveoli, hyperplasia and metaplasia of bronchial epithelial cells, mucus plugs defined as mucus material in bronchi and total inflammatory cells). The total inflammatory cell parameter was established by scoring infiltration of inflammatory cells into lung tissue and specific infiltration of peribronchial and perivascular inflammatory cells separately. Total histologic score results are mean ± SEM. The higher the histologic score, the more impaired the lung (8–16 mice/group, 3–14 lung sections/mouse scored). CTRL: control diet group; n-3: (n-3) LC-PUFA diet group; WT: wild-type mice; ΔF508: CftrΔF508 mice. **(B)** Representative lung sections from CftrΔF508 mice fed with control **(a)** or (n-3) LC-PUFA **(b)** diets after LPS challenge showing PAS positive mucus plug (P, zoom) with inflammatory cells. **(C)** Representative lung sections stained with HE 24 h after PBS (control) or LPS challenge. Black arrows outline collapsed alveoli; asterisks show inflammatory cells; red arrow heads indicate metaplasia; blue arrow heads indicate hyperplasia. Lu: lumen of bronchi.

Lung damage was evaluated in adult mice by two observers blinded to the genotype, diet and treatment, according to a homemade histologic score (see Methods). Weighted kappa was 0.73 ([0.51–0.95]; *P* = 0.002) between the two observers indicating substantial agreement [16]. Under basal conditions, CftrΔF508 mice given the control or (n-3) LC-PUFA enriched diet exhibited a 10-fold and 3-fold higher total histologic score, respectively, than WT mice (*P*<0.010; Fig 3 and Table 2). Injured areas in the lung (*P* = 0.0002), collapsed alveoli (*P* = 0.0004), hyperplasia of bronchial epithelial cells (*P* = 0.010) and infiltration of inflammatory cells (*P* = 0.007) mainly accounted for this difference. The increased hyperplasia of bronchial epithelial cells in CftrΔF508 mice fed the control diet compared to WT mice was not significant when CftrΔF508 mice received the (n-3) LC-PUFA diet. This shows that lungs of the CftrΔF508 mice exhibited a lung phenotype and (n-3) LC-PUFA diet does not exhibit any effect on adult CftrΔF508 mice.

**Table 2:** Effects of LPS on histological score according to mouse genotype and diet.

Genotype	Wild-type						CftrΔF508					
Diet	Control			(n-3) LC-PUFA			Control			(n-3) LC-PUFA		
	PBS	LPS	x(p)^a^	PBS	LPS	x(p)	PBS	LPS	x(p)	PBS	LPS	x(p)
Total score	**0.31±0.21**	**1.49±0.32**	4.8(***)	**1.04±0.34**	**2.14±0.41**	2.1(*)	**3.04±0.31**	**6.09±0.59**	2.0(***)	**3.14±0.42**	**6.45±0.62**	2.1(***)
Lung injury	**0.06±0.06**	**0.39±0.12**	6.5(*)	0.19±0.08	0.49±0.14	NS	0.88±0.18	0.90±0.09	NS	0.66±0.18	1.10±0.16	NS
Collapsed alveoli	**0.06±0.06**	**0.39±0.12**	6.5(**)	**0.19±0.08**	**0.48±0.13**	2.5(*)	0.86±0.18	0.90±0.09	NS	**0.63±0.18**	**1.10±0.16**	1.8(*)
Hyperplasia	0.00±0.00	0.00±0.00	NS	0.00±0.00	0.04±0.03	NS	**0.29±0.09**	**1.20±0.22**	4.2(**)	**0.14±0.10**	**0.83±0.21**	6.1(**)
Metaplasia	0.04±0.04	0.00±0.00	NS	0.06±0.06	0.02±0.02	NS	**0.16±0.07**	**1.20±0.17**	7.6(***)	**0.15±0.10**	**0.87±0.13**	5.8(***)
Mucus plugs	0.10±0.10	0.06±0.06	NS	0.07±0.07	0.13±0.09	NS	**0.00±0.00**	**0.50±0.19**	(**)	0.20±0.13	0.55±0.16	NS
Inflammatory cells	**0.05±0.04**	**0.64±0.09**	12.8(***)	**0.52±0.19**	**0.98±0.20**	1.9(*)	**0.85±0.20**	**1.39±0.28**	1.6(*)	**1.37±0.14**	**2.01±0.22**	1.5(**)

^a^: fold (*P* value);

* p<0.05;

** p<0.01;

*** p<0.001.

(n-3) LC-PUFA: (n-3) long-chain polyunsaturated fatty acids; PBS: phosphate-buffered saline; LPS: *P*. *aeruginosa* lipopolysaccharide; NS: not significant.

Acute lung inflammation was induced 24 h after inhalation of *P*. *aeruginosa* LPS. Concerning WT mice, LPS-acute lung inflammation increased the lung histologic score from 0.31±0.21 to 1.49±0.32 in mice fed the control diet (*P* = 0.004; Fig 3). The scores of WT mice fed the (n-3) LC-PUFA diet and challenged with PBS or LPS were not significantly different. CftrΔF508 mice from the two diet groups exhibited a higher histologic score when they had inhaled LPS (*P*<0.001) compared to PBS mice. Whatever the diet, the increase in score was much higher for CftrΔF508 mice (*P*<0.0001) and this increase was essentially due to increased hyperplasia of bronchial epithelial cells (*P* = 0.001 and *P* = 0.008 for control and (n-3) LC-PUFA groups, respectively; Table 2), metaplasia of Club cells (*P*<0.0001 and *P* = 0.0001 for control and (n-3) LC-PUFA groups, respectively) and infiltration of inflammatory cells (*P* = 0.035 and *P* = 0.004 for control and (n-3) LC-PUFA groups, respectively). LPS challenge increased the presence of mucus in the bronchi of CftrΔF508 mice fed the control diet (*P* = 0.0096; Table 2). The (n-3) LC-PUFA diet was associated with less metaplasia of Club cells in CftrΔF508 mice (*P* = 0.032; Fig 3) but also an increase in immune cell infiltration (*P* = 0.011). These data show that (n-3) LC-PUFA improved several histology parameters in the lungs of WT and CftrΔF508 mice.

### (n-3) LC-PUFA modulates lung inflammation

**Fig 4:**
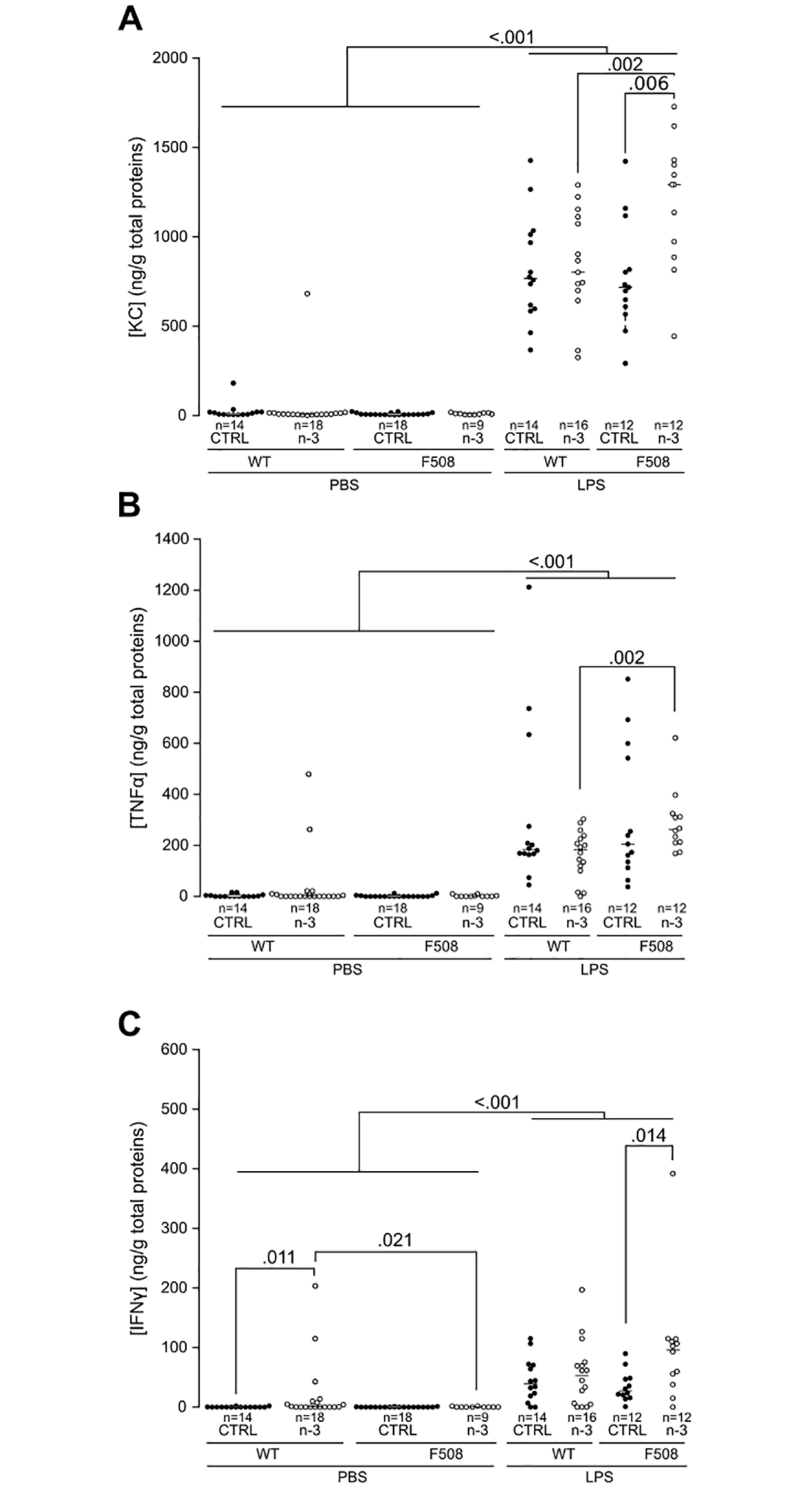
Detection of pro-inflammatory cytokines in lung homogenates by ELISA. **(A)** KC, **(B)** TNF-α and **(C)** IFNγ. Pro-inflammatory cytokine levels in wild-type (WT) and CftrΔF508 (ΔF508) mice were normalized to the concentration of total proteins (9–18 mice/group). CTRL: control diet group; n-3: (n-3) LC-PUFA diet group; WT: wild-type mice; ΔF508: CftrΔF508 mice.

The levels of KC, a chemokine (CXCL1) which attracts neutrophils, and the two proinflammatory cytokines TNF-α and IFNγ were studied by ELISA in lung homogenates of adult mice. Without any inflammatory challenge, the cytokine levels remained very low (Fig 4A–4C). We only noticed a mild increase in IFNγ in WT mice fed (n-3) LC-PUFA (*P* = 0.011, Fig 4C).

LPS induced a dramatic increase in the three cytokines within 24 h (*P*<0.001, Fig 4A–4C). The (n-3) LC-PUFA diet was associated with higher levels of KC and IFNγ in CftrΔF508 mice when compared to CftrΔF508 mice fed the control diet (*P* = 0.006 and *P* = 0.014, respectively).

**Fig 5:**
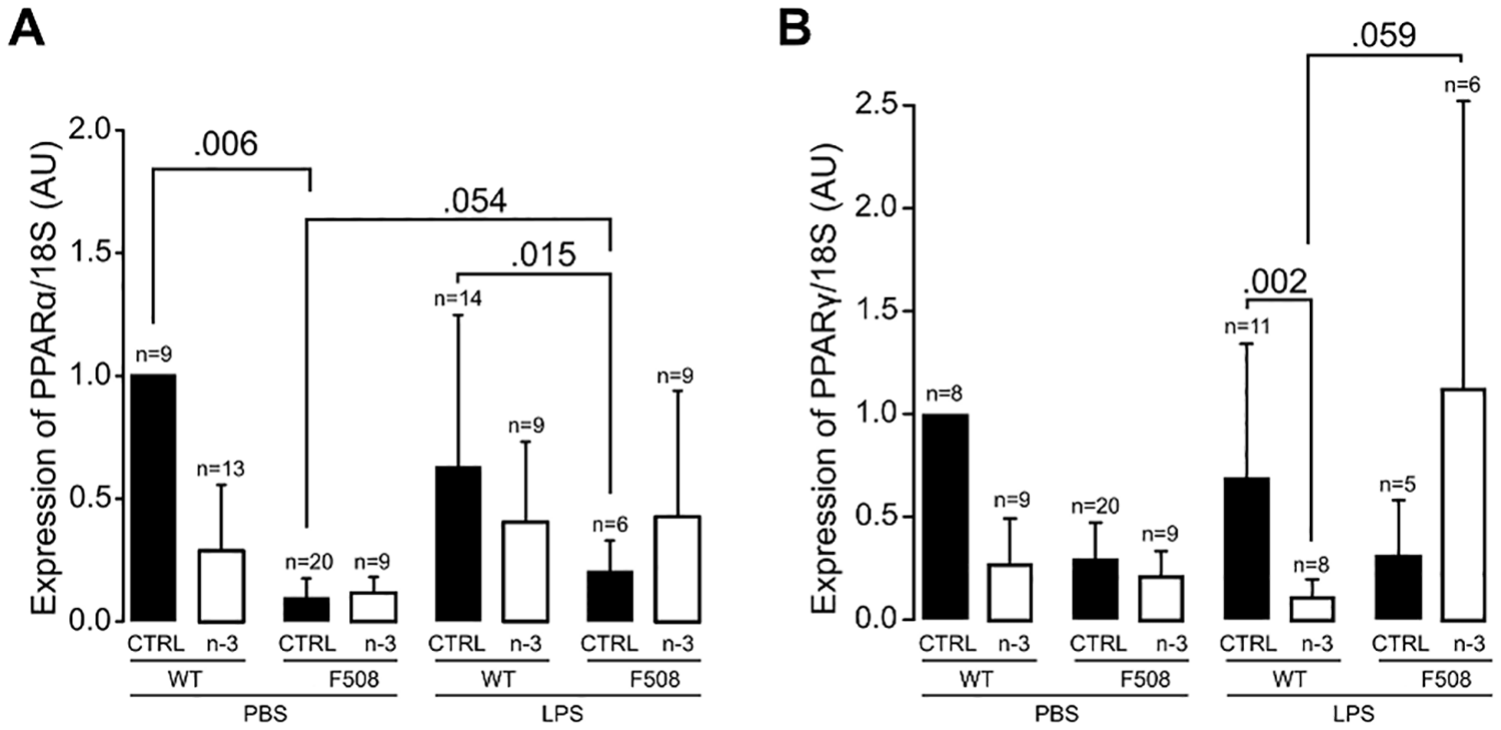
PPAR expression in lung homogenates by RT-qPCR. **(A)** *PPARα* and **(B)** *PPARγ*. Transcripts were normalized to *18S*. Data shown are mean ± standard error (5–20 mice/group). CTRL: control diet group; n-3: (n-3) LC-PUFA diet group; WT: wild-type mice; ΔF508: CftrΔF508 mice.

We next studied the effects of the (n-3) LC-PUFA diet on gene expression of the two markers of resolution of inflammation, PPARα and PPARγ, in lung homogenates. Transcript levels of the two genes were similar before and after LPS challenge with the exception of a tendency (*P* = 0.054) for an increase in PPARα in CftrΔF508 mice fed the control diet. PPARα but not PPARγ mRNA levels were significantly higher in the control diet group of unchallenged and LPS-challenged WT mice compared to CftrΔF508 mice (*P* = 0.006 and *P* = 0.015, respectively; Fig 5A). The (n-3) LC-PUFA diet was associated with a significant decrease in PPARγ expression in WT mice (*P* = 0.002) but not in CftrΔF508 mice (Fig 5B). Together these data show that (n-3) LC-PUFA modulates an early inflammatory response in the lungs after LPS challenge in both WT and CftrΔF508 mice.

## Discussion

We have previously evaluated an isocaloric/isolipidic (n-3) LC-PUFA supplementation upon adult WT and Cftr^–/–^ mice infected chronically or acutely with *P*. *aeruginosa*. Five weeks administration of the (n-3) enriched diet decreased mortality and gel-forming Muc5b overexpression of infected WT mice, an increased distal alveolar fluid clearance and induced an increase in neutrophil flux and TNF-α release 24 hours post-infection (reviewed in Husson *et al*. [17]). We reported an improved lung injury and survival, accelerated bacterial clearance, and decreased inflammation in C57BL/6 mice and improved the outcome of acute *P*. *aeruginosa* infection in Cftr^–/–^ mice. In the present study we used CftrΔF508 mice and LPS instillation at day 60 of age. We found that CftrΔF508 mice exhibit growth retardation at PN21 which is corrected by a (n-3) LC-PUFA diet throughout adulthood, especially in males. The growth retardation observed is in agreement with previous published studies showing that adult Cftr^–/–^ and Cftr^ΔF508/ΔF508^ mice have a reduced body mass [17,18]. In humans, growth retardation is a hallmark of CF in children [19] and is correlated with the severity of lung disease [20,21]. The growth deficit is usually related to lipid malabsorption which leads to energy deficit [22,23]. One hypothesis to explain the beneficial long-term effect of the (n-3) LC-PUFA diet is that the lower inflammatory status and reduced lung damage leads to a lower energy expenditure.

According to the three cytokines studied, no difference was observed in inflammatory status between unchallenged WT and CftrΔF508 mice. However, the lungs of CftrΔF508 mice were found to exhibit a higher histologic score than WT mice due to the combination of an increased inflammatory cell infiltration with collapsed alveoli and injured areas. This is in agreement with constitutive inflammation of CftrΔF508 mice characterized by neutrophil accumulation in the lungs and release of the pro-inflammatory cytokine MIP-2 [24,25], which is a mouse analog of human IL-8. Indeed, CftrΔF508 mice are known to display multiple inflammatory mediators and immune cells in lung tissue [26]. The mouse situation is similar to CF children who secrete more neutrophil chemotactic factor IL-8 than non-CF children with bacterial infection of the lower airways [27]. Neutrophil chemotactic factor KC has been suggested to be an important mediator of the enhanced inflammatory response observed in lungs from Cftr^–/–^ mice [28]. We did not observed any variation in KC between WT and CftrΔF508 mice fed the control diet but this difference may arise from the different mouse models studied, genetic background, nutrition, animal facility and timing of dosage/kinetics of inflammatory markers.

Our histologic score was built from seven well-characterized criteria of CF lung abnormalities in unchallenged CftrΔF508 mice as reported previously in independent studies [25,29]. Such spontaneous lung abnormalities in CF mouse models are controversial in the literature and seem to depend on many factors including genotype, genetic background, age and environment of the mice [30,31]. CftrΔF508 mice exhibited an exacerbated inflammatory response to *P*. *aeruginosa* LPS in agreement with previous studies in Cftr^–/–^ and CftrΔF508 mouse models [24,32–34].

CF patients and CF mouse models are more susceptible to infection of the airways with opportunistic bacteria such as *P*. *aeruginosa* and display excessive inflammation which may lead to lung fibrosis [35]. The beneficial effects of the (n-3) LC-PUFA diet in CftrΔF508 mice after LPS challenge that we observed are the decrease in lung metaplasia and modulation of the inflammatory response as shown by the significantly lower levels of PPARα in CftrΔF508 mice fed the control diet counterbalanced for CftrΔF508 mice fed the (n-3) LC-PUFA diet (compared to WT mice, Fig 5A). However, it is important to note that the levels of PPAR expression determined here must be considered as low. The main reason for this is that 24 h after lung acute inflammation is probably too late to observe the peak in PPAR expression [12]. The increased cytokines and inflammatory cells of CftrΔF508 mice fed the (n-3) LC-PUFA diet we found in our model could potentially be worse than beneficial and this is not in agreement with Freedman *et al*. who reported that 7 days of orally administrated DHA to Cftr^−/−^ mice which are further exposed to aerosolized Pseudomonas LPS daily for 3 days blocked the neutrophil recruitment in BAL [36]. However, the effects of (n-3) LC-PUFA diet on the inflammatory kinetic greatly depend on the n-3 LCPUFA dose and timing [17]. Furthermore, it is possible that the higher increased of cytokines and neutrophils recruitment 24 hours after LPS instillation may help mice to resolve the inflammatory process.

The (n-3)/(n-6) LC-PUFA imbalance found in CF mouse models and CF patients may exacerbate the inflammatory response reported in both mice and CF patients [36,37] as anti-inflammatory pathways in the lung are in part mediated by PPARα and γ inhibiting cytokine production [38,39]. Indeed, lower levels of PPARα have been reported in CF patients [40]. This has led to clinical trials in which CF patients were supplemented with (n-3) LC-PUFA [9,41]. The evidence is currently insufficient to draw firm conclusions or recommend the routine use of (n-3) supplements in CF patients. In fact, the source of (n-3) fatty acids, dosage, duration, age of patients, parameters measured and CFTR genotype are too diverse between studies. Animal models may greatly help by reducing inter-individual genotype variability in a controlled environment. A higher ratio of (n-3)/(n-6) LC-PUFA in cells may represent a good alternative to decrease the pro-inflammatory status and to limit the overproduction of pro-inflammatory molecules. Dietary supplementation of a low dose of DHA for 6 weeks has been shown to increase the DHA/AA ratio in CftrΔF508 mice [10]. However, because the imbalance in (n-3)/(n-6) LC-PUFA ratio in cells starts early in the life of CF patients and mice [42,43], we chose to supplement the mice with EPA+DHA for the longest possible time at a dose that can be extrapolated to human consumption. This leads to a higher (EPA+DHA)/AA ratio in the liver and lung tissues of CftrΔF508 pups via enrichment of the milk of the mother and/or possibly during fetal development. This suggests that early dietary supplementation of pregnant mothers with (n-3) LC-PUFA may improve lung function in children with CF.
